# Regulatory Emotional Self‐Efficacy and Hedonic Well‐Being in Daily Life

**DOI:** 10.1002/ijop.70123

**Published:** 2025-11-17

**Authors:** Antonio Zuffianò, Fulvio Gregori, Lucia Manfredi, Elisabetta Beolchini, Silvia Caldaroni, Alessia Teresa Virzì, Noemi Di Brango, Virginia Isabel Barrero Toncel, Stefania Sette, Valentina Paz Quilodrán Fuentes, Bernadette Paula Luengo Kanacri

**Affiliations:** ^1^ Sapienza, University of Rome Rome Italy; ^2^ Pontificia Universidad Catόlica de Chile Santiago de Chile Chile

**Keywords:** dynamic structural equational model, hedonic well‐being, intensive longitudinal data, regulatory emotional self‐efficacy

## Abstract

Successfully managing one's unpleasant emotions despite adversities is important to help people maintain their well‐being. Using daily diary assessment, we explored the role of Self‐efficacy in Regulating Negative Affect (SRN) on Hedonic well‐being (HWB), measured once a day over 21 days in a sample of 63 Italian young adults (mean age = 25.43, SD = 3.47; 67.7% women). Dynamic Structural Equation Modelling showed that there is a significant positive correlation between the spill‐over effect from daily SRN_*t*−1_ to HWB_*t*_, with the carry‐over effect of SRN (*r* = 0.625; 95% CI: [0.179, 0.850]). To explore this result more in depth, we performed a simple slope analysis that revealed that for those young adults with high carry‐over of SRN over time, the spill‐over effect from daily SRN_*t*−1_ to HWB_*t*_ was positive and statistically significant (*b* = 0.247, 95% CI [0.032, 0.447]) compared to those young adults with low carry‐over of SRN, which was nonsignificant (*b* = −0.092, 95% CI [−0.299, 0.137]). We discuss these findings in light of the potential role that SRN could play in people's well‐being during their everyday lives, which is in line with Bandura's social cognitive theory. At the practical level, our results suggest that enhancing SRN might be beneficial to young adults' HWB.

People's ability to regulate their negative emotions is an important aspect of their social functioning and well‐being (Nyklíček et al. 2011). For instance, the consequences of being unable to manage one's anger do not only concern difficulties in interpersonal relationships (e.g., heightened chances of aggressive reactions leading to social rejection) but are also related to physiological issues (e.g., blood pressure and hypertension) that, in the long run, could compromise people's health at different levels (Staicu and Cuţov 2010). Similarly, people's difficulties in managing their anxiety and sadness could undermine their ability to cope with daily tasks (e.g., at work), leading to a detrimental spiral of negative states (e.g., Feng et al. 2023).

Within this area of research, several personality psychologists (e.g., Alessandri et al. 2022; Bassi et al. 2018; Caprara et al. 2013) have focused on regulatory emotional self‐efficacy, namely the beliefs people hold about their capability to master their negative emotions (SRN). From a social‐cognitive perspective (Bandura 2001), people are seen as active agents who reflect upon their experiences and try to exert control in different areas of their life, including the emotional domain. People who feel capable of managing their unpleasant emotions are indeed more likely to succeed in their regulatory efforts and persist in such efforts despite possible drawbacks (e.g., people's capability to get over irritation quickly for wrongs they have experienced). Importantly, recent meta‐analytic findings (Alessandri et al. 2022) confirmed the protective role of regulatory emotional self‐efficacy beliefs both against maladjustment (e.g., antisocial behaviour, externalising problems) and in favour of positive outcomes (e.g., health, well‐being).

Considering the positive effect of SRN on hedonic well‐being (HWB; Kahneman et al. 1999)—namely people's subjective evaluation of their happiness (emotional component) and life satisfaction (cognitive component), several scholars have focused on inter‐individual (between‐person) differences. However, less attention has been paid to how SRN may enhance well‐being in the short term, particularly on a daily basis. Adopting such an approach can provide a more precise understanding of how daily fluctuations in SRN influence HWB at the intra‐individual (within‐person) level, capturing the dynamic nature of these processes over time (Kuper et al. 2024).

In light of these considerations, the present work aims to investigate the effect of SRN on HWB in a sample of Italian young adults (18–35 years; Arnett 2000) followed daily over 21 days (i.e., 3 weeks). Based on recent recommendations for drawing causal inference in psychological research (i.e., Rohrer and Murayama 2023), we focused on the within‐person effects of SRN on HWB (i.e., higher‐than‐usual levels of SRN as predictors of higher‐than‐usual levels of HWB) by separating them from between‐person associations that might represent stable, time‐invariant confounders of such relations. To maintain the between/within‐person distinction, we used Dynamic Structural Equation Modelling (DSEM; Hamaker et al. 2018), a novel technique that combines time series analysis and multilevel modelling to analyse intensive longitudinal data (i.e., 20 or more waves of data) collected through daily diaries (Bolger et al. 2003).

## 1 SRN and HWB

People who feel capable of managing their negative emotions perceive themselves as exerting control (at least to a certain extent) over the causes of their emotions, their expression and their possible consequences (Caprara et al. 2013). The perception of agency over one's unpleasant emotions, indeed, allows people to experience HWB since they feel they have the resources to cope with the challenges in their everyday life; they likely tend to rely less on maladaptive regulation strategies (e.g., rumination), as well as they are more competent in social interactions leading them to higher satisfaction with their life (e.g., Caprara and Steca 2005a; Lightsey Jr. et al. 2011).

The empirical evidence has largely corroborated the positive role of SRN in people's HWB in both cross‐sectional and longitudinal studies. For instance, in a cross‐sectional study with university students, Lightsey Jr. et al. (2011) found that SRN buffered the negative effect of negative affect on life satisfaction (i.e., among young adults with high SRN [+1SD], the effect of negative affect on life satisfaction was nonsignificant whereas it was negative and statistically significant among young adults with low [−1SD] and medium [*mean*] levels of SRN). The positive effect of SRN on life satisfaction and hedonic balance (i.e., people's higher experience of positive emotions than negative emotions) has also been found in studies using complex mediational mechanisms linking SRN to the specific facets of HWB (e.g., Caprara and Steca 2005a, 2005b; Caprara et al. 2006).

Longitudinal evidence has been provided by Lightsey Jr. et al. (2013) who found that, after controlling for initial levels of life satisfaction, higher levels of self‐efficacy in regulating despondency and distress predicted greater life satisfaction after 17 days (*β* = 0.21; *p* < 0.01). Self‐efficacy in managing anger, instead, did not predict later life satisfaction (*β* = 0.02; *p* = 0.75). Interestingly, the protective role of SRN has also been highlighted in short‐term, intensive longitudinal studies. For instance, Bassi et al. (2018) asked more than 200 Italian adolescents to report—eight times a day over 7 days—their positive and negative emotions. Multilevel results showed that—throughout the study—those adolescents with higher SRN (measured at the baseline) also felt happier (*β* = 0.14, *p* < 0.01) and experienced less sadness (*β* = −0.12, *p* < 0.05) compared to their counterparts. In a very recent study, Kleiman et al. (2023) collected information on both negative emotions and SRN four times a day over 6 weeks from 145 college students. The results of their study indicated that SRN was negatively associated with unpleasant emotions both in terms of stable inter‐individual differences (between‐person level) and momentary fluctuations (within‐person levels), thereby suggesting the importance of taking into account both levels of analysis.

## 2 The Present Study

Following recent studies focusing on the role of SRN in everyday life (e.g., Bassi et al. 2018; Kleiman et al. 2023; Zuffianò et al. 2023), in the present work, we investigated the effect of SRN on HWB in a sample of 63 young adults followed once a day over 3 weeks. Specifically, we focused on self‐efficacy in regulating sadness and anger because they are the most frequent negative emotions in people's daily lives (Trampe et al. 2015). Furthermore, anger represents the prototypical high‐arousal emotion (Phillips et al. 2006), while sadness is the prototypical low‐arousal negative emotion (Rivers et al. 2007). Therefore, considering self‐efficacy in regulating these two emotions allowed us to capture the full spectrum of negative emotions more effectively. To separate the effects at the within‐person level (i.e., momentary fluctuations in SRN predicting momentary fluctuations in HWB) from between‐person variability, we used DSEM, a novel technique that not only distinguishes the two levels of analysis but also permits the estimation of within‐person effects as random effects, thereby allowing the researchers to unravel potential between‐person differences (inter‐individual variability) in the within‐person effects. As noted by Hamaker (2012), understanding the within‐person dynamics among the variables of interest might shed light on the micro‐processes (e.g., peaks in SRN causing peaks in HWB) that, in the long run, could lead to future between‐person differences. Moreover, since both SRN (e.g., Zuffianò et al. 2023) and HWB (Christodoulou et al. 2014) could be affected by daily events—especially among young adults—we tested the effects of SRN on HWB while controlling for age and gender. Additionally, we also controlled for possible major negative events such as health issues, conflicts with others and economic difficulties.

## 3 Method

### 3.1 Samples and Procedure

The present study is part of a broader intensive longitudinal project aimed at examining risk and protective factors for young adults' well‐being in daily life (e.g., Caldaroni et al. 2025). The sample of the present study included 63 young adults from Italy (18–35 years old; mean age = 25.43, SD = 3.47; 67.7% women). Regarding education, 38.1% have completed their secondary studies. Participants were either full‐time workers (27%), full‐time university students (23.8%) or both university students and workers. In terms of relationship status, 49.2% were single and more than half (52.4%) identified as agnostic or atheist. Additionally, 44% of the participants reside in large population centers. Their annual household income ranges between €16,000–29,000 (23.8%) and €30,000–40,000 (22.2%).

In line with the recommendations of Maas and Hox (2005) for nested data, our goal was to recruit more than 50 participants for our study.^1^ The Ethics Committee approved the study at the author's institution. Researchers recruited participants with different backgrounds and outlooks through the snowball procedure, as suggested by Parker et al. (2019). All participants were invited to fill out a baseline questionnaire to obtain socio‐demographic data and other individual characteristics such as personality traits and self‐efficacy beliefs, after giving their informed consent. Participants received daily short online questionnaires (each questionnaire took approximately 4–5 min to be completed) each day across 21 days. The daily data were collected during the evening from 8 PM to 11:59 PM. Participants received a gift card for a bookstore for a maximum value of 25€ as a token of appreciation for their time. In terms of the response rating, 3.2% of adults completed 7 or fewer daily diaries, 14.3% completed between 7 and 14 daily diaries and 82.5% completed between 14 and 21 diaries.

### 3.2 Daily Measures

#### 3.2.1 Daily Self‐Efficacy in Managing Negative Emotions (SRN_DL)

Participants rated (from 1 = *not well at all* to 5 = *very well*) their momentary self‐efficacy beliefs in managing their despondency and anger (i.e., Right now, how well can you keep from getting discouraged in the face of difficulties?; Right now, how well can you control your anger when you are treated badly and unfairly?). The items were adapted from the Regulatory Emotional Self‐Efficacy scale (RESE; Bandura et al. 2003; Caprara et al. 2013) to capture the regulatory self‐efficacy beliefs day to day. The score was obtained by averaging the two adapted items.

#### 3.2.2 Hedonic Well‐Being (HWB)

The score was obtained by averaging two adapted items capturing participants' happiness (PANAS; Watson et al. 1988) and life satisfaction (from SWLS; Diener et al. 1985). The items were ‘Today, to what extent did you feel happy?’ on a 5‐point Likert scale (1 = *not at all*; 5 = *extremely*) and ‘Today I was satisfied with my life’ on a 5‐point Likert scale (1 = *strongly disagree*; 5 = *strongly agree*).

#### 3.2.3 Negative Daily Events (N_DLE)

Participants reported whether certain negative life events occurred during their day (Gable et al. 2000), such as conflict with other people, problems related to work or study, economic difficulties or health problems, using a Likert scale from 0 (*it didn't happen*) to 2 (*it happened and was of great importance*).

### 3.3 Data Analytic Approach

**FIGURE 1:**
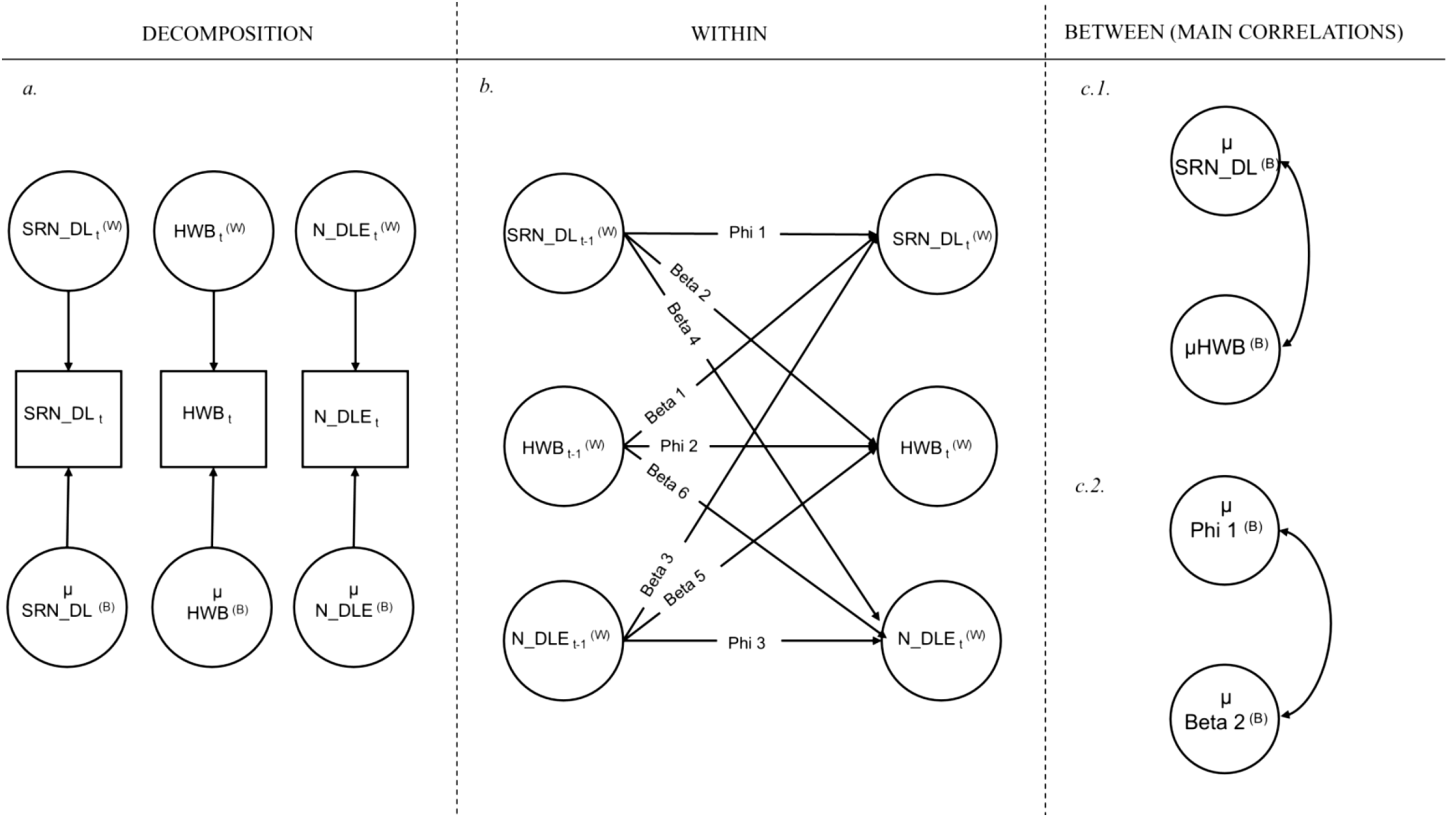
Beta 1, spill‐over from HWB to SRN_DL; Beta 2, spill‐over from SRN_DL to HWB; Beta 3, spill‐over from N_DLE to SRN_DL; Beta 4, spill‐over from SRN_DL to N_DLE; Beta 5, spill‐over from N_DLE to HWB; Beta 6, spill‐over from HWB to N_DLE; HWB, hedonic well‐being; N_DLE, negative daily events; Phi 1, carryover of SRN_DL; Phi 2, carryover of HWB; Phi 3, carryover of N_DLE; SRN_DL, daily self‐efficacy in regulating negative emotion. The left figure (a) shows the decomposition of the within‐person (time‐varying) and between‐person level (time‐invariant). The middle figure (part b) shows the lagged effects between SRN_DL, HWB and N_DLE. The part on the right shows the between‐person level correlations among SRN_DL and HWB (c.1.) and between PHI1 and BETA2 (c.2.).

To analyse daily data we used DSEM in M*Plus* 8.10 (Muthén and Muthén 1998–2023), a technique that comprises both time series analysis (when *N* = 1) and multilevel variations of this when *N* > 1 (Hamaker et al. 2018; McNeish and Hamaker 2020). In the DSEM framework, the within‐person level of a variable (intra‐individual changes) and the between‐person level (stable inter‐individual differences) can be clearly disentangled, and, therefore, the dynamics across the constructs of interest (i.e., the within‐person effects) can be studied while controlling for stable inter‐individual differences in the same variables (Hamaker et al. 2018). At the within‐person level, the DSEM includes: (i) carryover effects, which refer to the autoregression parameter of the considered variable on itself at the previous time point (*t*−1) and reflect the persistence of the variables of interest (e.g., the extent to which peaks in HWB tend to persist over time). The carry‐over effect captures how quickly a person returns to equilibrium after being disrupted and it is also known as inertia. The carryover effect typically ranges from 0 to 1 and values near 1 reflect strong persistence (Hamaker et al. 2018); (ii) the spill‐over effects, which capture the regression of one variable on another measured previously (see Figure 1) and are especially interesting because they may represent potential causal mechanisms, indicating the cascade effect of functioning or behaviour from one domain into another (Hamaker et al. 2018); (iii) the time‐specific within‐person correlations between the variables of interest, reflecting their simultaneous, occasion‐specific associations. Both carryover and spill‐over effects were treated as random (to capture the variability in their strength across participants) and they were allowed to covary among themselves as well as with the random components of the intercepts of HWB, SRN_DL and N_DLE at the between‐person level (i.e., the participants' general level of HWB, SRN_DL and N_DLE across the 21 days; see Figure 1). Given the presence of a large number of random effects (which would be intractable in frequentist statistics; Hamaker et al. 2018), the DSEM adopts a Bayesian estimation of the parameters. At the between‐person level, DSEM permits the estimation of the intercepts (means) of the variables of interest and the correlations among the random effects.

We ran the unconditional model as per the guidelines provided by Hamaker et al. (2018). First, we performed the unconditional model 1 in which our variables of interest, SRN_DL, HWB and N_DLE reciprocally influenced each other across 21 days. In this model, the within‐person effects (i.e., spill‐over and carry‐over effects) were treated as ‘random’ as they were allowed to vary among participants. We also assessed the time‐specific within‐person correlations between SRN_DL, HWB and N_DLE. Furthermore, as reported in Figure 1 we investigated the between‐level correlations of the intercepts (i.e., average levels across the 21 days) of SRN_DL, HWB and N_DLE. We also investigated the between‐level correlations among the intercepts and the random effects. We ran our DSEM with 3000 iterations. To evaluate the statistical significance of the effects, we considered 95% credible intervals (95% CI). For model adequacy indices, the Potential Scale Reduction (PSR) is frequently used in Bayesian models, as it provides a reliable measure of convergence. We utilised the PSR to ensure model convergence, as recommended by Hamaker et al. (2018) and Asparouhov and Muthén (2023), who suggest that the PSR should be approximately 1.

## 4 Results

### 4.1 DSEM

Syntaxes, outputs, and the anonymized dataset are uploaded in the OSF at the following link: https://osf.io/vfyzj/?view_only=8dab6c94123240d2962a910330c276a6.

Regarding the Intraclass Correlation Coefficient (ICC), the values for each outcome variable are as follows: SRN_DL = 0.51, HWB = 0.40 and N_DLE = 0.37.

The Potential Scale Reduction indices (PSR) indicate that all DSEM models have converged adequately, with the highest index value being 1.109.

The unconditional DSEM (model 1; i.e., in which we included SRN_DL, HWB and N_DLE) showed at the between‐person level some significant correlations among the intercepts of the variables and the random effects (see Table 1). Specifically, there was a positive correlation between SRN_DL and HWB, indicating that, on average, people who reported higher values of SRN_DL also reported higher HWB than their counterparts (*r* = 0.551; 95% CI: [0.190, 0.775]). Moreover, the means of SRN_DL and HWB were high during the 21 days (M_SRN_DL_ = 4.503; 95% CI: [3.342, 5.923]; M_HWB_ = 4.748; 95% CI: [3.535, 6.278]; scores ranged from 1 to 5).

**TABLE 1:** Between‐person correlations from the unconditional DSEM at 3000 iterations.

Parameters	Between‐level standardised estimates		
	95% CI		
	Estimate	Lower	Upper
SRN_DL ↔ HWB	0.551	0.190	0.775
HWB ↔ N_DLE	−0.459	−0.707	−0.121
PHI1 ↔ BETA2	0.625	0.179	0.850
PHI2 ↔ BETA1	0.602	0.125	0.853
PHI3 ↔ BETA5	−0.551	−0.828	−0.007
BETA4 ↔ BETA6	−0.550	−0.835	−0.001

*Note:* For the sake of simplicity, we reported only the significant correlations. For the full results, please see the OSF LINK https://osf.io/vfyzj/?view_only=8dab6c94123240d2962a910330c276a6.

Abbreviations: BETA1, Spill‐over effect from HWBT_*t*−1_ to SRN_DL_*t*_; BETA2, Spill‐over effect from SRN_DL_*t*−1_ to HWB_*t*_; BETA4, Spill‐over effect from SRN_DL_*t*−1_ to N_DLE_*t*_; BETA5, Spill‐over effect from N_DLE_*t*−1_ to HWB_*t*_; BETA6, Spill‐over effect from HWB_*t*−1_ to N_DLE_*t*_; CI, 95% credible intervals; DSEM, dynamic structural equation modelling; HWB, hedonic well‐being; N_DLE, negative daily events; PHI1, carry‐over of SRN_DL; PHI2, carry‐over of HWB; PHI3, carry‐over of N_DLE; SRN_DL, daily self‐efficacy in regulating negative emotions.

At the within‐person level, the DSEM showed positive and significant carryover effects of SRN_DL (PHI1), *β* = 0.194 (95% CI [0.106, 0.283]), HWB (PHI2) *β* = 0.116 (95% CI [0.030, 0.210]) and N_DLE (PHI3) *β* = 0.155 (95% CI [0.068, 0.240]; see Table 2). The spillover effects and within‐person correlations among the variables of interest are reported in Table 2. DSEM showed only one statistically significant spillover effect from N_DLE_*t*−1_ to HWB_*t*_ (BETA5), indicating that higher‐than‐expected levels in N_DLE at a given day predicted lower‐than‐expected levels in HWB on the subsequent day (*β* = −0.088, 95% CI [−0.167, −0.006]; see Table 2). Interestingly, although the average spillover effect from SRN_DL_*t*−1_ to HWB_*t*_ (BETA2) was not statistically significant (*β* = 0.070, 95% CI [−0.003, 0.155]), it showed significant variability (*σ* ^2^ = 0.280, 95% CI [0.123, 0.574]), indicating interindividual differences in the strength of such effect. This spillover effect from SRN_DL_*t*−1_ to HWB_*t*_ (BETA2) was, indeed, positively correlated with the carryover effect of SRN_DL (PHI1), meaning that people who had high levels of carryover of SRN_DL (PHI1) also reported a stronger spillover effect from SRN_DL_*t*−1_ to HWB_*t*_ (*r* = 0.625; 95% CI: [0.179, 0.850]; see Table 1). To examine more in depth this result, we followed a three‐step approach: firstly, we used the command ‘(CLUSTER) FS COMPARISON’ on Mplus to obtain subject‐specific standardised PHI1 estimate; secondly, we created a dichotomous variable ‘low and high PHI1’ (L_H_PHI1) by assigning participants to the ‘low PHI1’ group (value 0) if their PHI1 was below the group mean (0.194) and to the ‘high PHI1’ group (value 1) if their PHI1 was above the group mean; thirdly, we ran a conditional DSEM (2.a), in which we considered L_H_PHI1 as a predictor of the intercepts of the variables of interest and BETA2 (see Table 3).

**TABLE 2:** Within‐person results from the unconditional DSEM at 3000 iterations.

Parameters	Within‐level standardised estimates		
	95% CI		
	Estimate	Lower	Upper
Carryover effects			
SRN_DL_*t*−1_ → SRN_DL_*t*_ (PHI1)	0.194^a^	0.106	0.283
HWB_*t*−1_ → HWB_*t*_ (PHI2)	0.116^a^	0.030	0.210
N_DLE_*t*−1_ → N_DLE_*t*_ (PHI3)	0.155^a^	0.068	0.240
Spill‐over effects			
HWB_*t*−1_ → SRN_DL_*t*_ (BETA1)	0.078	−0.014	0.168
SRN_DL_*t*−1_ → HWB_*t*_ (BETA2)	0.070	−0.003	0.155
N_DLE_*t*−1_ → SRN_DL_*t*_ (BETA3)	−0.028	−0.106	0.054
SRN_DL_*t*−1_ → N_DLE_*t*_ (BETA4)	−0.028	−0.114	0.060
N_DLE_*t*−1_ → HWB_*t*_ (BETA5)	−0.088^a^	−0.167	−0.006
HWB_*t*−1_ → N_DLE_*t*_ (BETA6)	−0.045	−0.143	0.050
Within‐person correlations			
HWB ↔ N_DLE	−0.405^a^	−0.460	−0.345
SRN_DL ↔ N_DLE	−0.301^a^	−0.359	−0.238
SRN_DL ↔ HWB	0.483^a^	0.430	0.532

Abbreviations: CI, 95% credible intervals; DSEM, dynamic structural equation modelling; HWB, hedonic well‐being; N_DLE, negative daily events; SRN_DL, daily self‐efficacy in regulating negative emotions.

^a^ Credible interval did not include zero.

**TABLE 3:** Results from the conditional 2.a and 2.b DSEM at 3000 Iterations.

Parameters	Within‐level standardised estimates		
	95% CI		
	Estimate	Lower	Upper
Conditional 2.a			
L_H_PHI1 → BETA2	0.372^a^	0.073	0.649
L_H_PHI1 → N_DLE	−0.159	−0.402	0.124
L_H_PHI1 → SRN_DL	0.322^a^	0.034	0.547
L_H_PHI1 → HWB	0.366^a^	0.091	0.574

Abbreviations: BETA2, spill‐over effects from SRN_DL and HWB; CI, 95% credible intervals; DSEM, dynamic structural equation modelling; HWB, hedonic well‐being; L_H_PHI1, low and high levels of PHI1; N_DLE, negative daily events; SRN_DL, daily self‐efficacy in regulating negative emotions.

^a^ Credible interval did not include zero.

**FIGURE 2:**
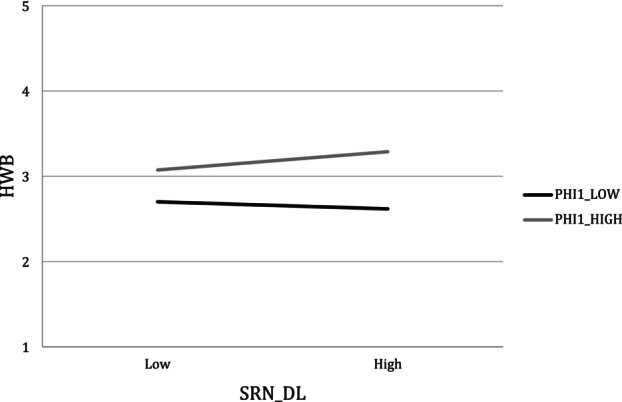
Simple slope of L_H_PHI1 on the within effect from SRN_DL to HWB. HWB, hedonic well‐being; PHI1_HIGH, high level of the carry‐over of self‐efficacy in regulating negative emotions; PHI1_LOW, low level of the carry‐over of self‐efficacy in regulating negative emotions; SRN_DL, daily self‐efficacy in regulating negative emotion.

The purpose of creating this dichotomous variable was to facilitate an exploratory analysis that clearly distinguished between individuals who reported low carryover from those with high carryover over the 21‐day period. Of importance, we found that L_H_PHI1 significantly predicted BETA2, indicating the presence of a cross‐level interaction (*β* = 0.372; 95% CI: [0.073, 0.649]).^2^ Simple slopes analysis (conditional DSEM 2.c) showed that for those young adults with high carryover of SRN_DL (*b* = 0.247, 95% CI [0.032, 0.447]; see Figure 2) the spillover effect from SRN_DL_*t*−1_ to HWB_*t*_ (BETA2) was positive and statistically significant compared to those young adults with low carryover of SRN_DL over time (i.e., weak PHI1; *b* = −0.092, 95% CI [−0.299, 0.137]).

Additional analyses conducted to check the robustness of the results are reported in Supporting Information.

## 5 Discussion

Successfully managing one's unpleasant emotions despite adversities is important to help people maintain their well‐being. In the present study, we focused on people's SRN, namely their perceived capability to deal with their *despondency* in the face of difficulties and *anger* when treated unfairly in their everyday life. As explained by Bandura (2001), among the mechanisms of human agency, self‐efficacy beliefs are an essential part of human agency since people will not engage in any activities (including emotion regulation) if they do not feel capable of doing so. Hence, we reasoned that perceptions of being capable of exerting some control over one's unpleasant emotions could help people experience higher HWB because they also feel more agentic over their happiness and satisfaction with life. Moreover, people who do not feel capable of managing their negative emotions (e.g., anger) are more likely to engage in dysfunctional emotion regulation strategies (e.g., anger rumination) that could undermine people's positive interpersonal relationships, which are an important ingredient of people's well‐being (Lightsey Jr. et al. 2011). Similarly, a perceived incapability to deal with one's despondency in the face of difficulties could further exacerbate people's hopelessness and negative views of the world, which are key cognitive components of depression (Beck et al. 1987).

Although previous studies have already highlighted the positive role of SRN (e.g., Alessandri et al. 2022; Lightsey Jr. et al. 2011), the extent to which SRN could positively affect people's HWB during people's everyday life only recently has started to receive attention in the literature (see Bassi et al. 2018; Kleiman et al. 2023). Following this line of research, we employed intensive longitudinal data collected through daily diaries (Bolger et al. 2003) and analysed through DSEM (Hamaker et al. 2018) to unravel how day‐to‐day changes in SRN influenced daily fluctuations in HWB within individuals while controlling for the between‐person differences therein. Specifically, we followed 63 young adults once a day over 3 weeks to assess if those days in which participants reported higher SRN_DL than their usual were related to higher HWB than their usual both concurrently (on the same day) and longitudinally (the day after). In detail, we focused on the within‐person effects of SRN_DL on HWB while taking into account the occurrence of daily negative events that could compromise both SRN_DL and HWB as well as the stable, between‐person factors (e.g., trait‐like variability in SRN_DL and HWB) that could confound such effect (Rohrer and Murayama 2023).

Results from the DSEM—at the between‐person level—showed significant correlations among the general levels of SRN_DL and HWB, indicating that young adults with higher SRN_DL across the 3 weeks also reported higher HWB than their counterparts. This result is in line with previous cross‐sectional works attesting to the benefits of feeling capable of managing one's unpleasant emotions on HWB (e.g., Lightsey Jr. et al. 2011).

At the within‐person level (i.e., the focus of our analyses), the average spill‐over effect of SRN_DL on subsequent HWB was not statistically significant, indicating that, on average, feeling more capable than one's usual managing despondency and anger did not predict a peak (i.e., higher‐than‐usual levels) of happiness and life satisfaction the next day. Yet, this spill‐over effect was positively associated with the carry‐over of SRN_DL as attested by the statistically significant correlation between these two random effects. More in detail, those young adults who experienced higher persistence of their daily peaks in SRN_DL (i.e., the carry‐over effect captures the stability of such deviations/perturbations in the system) were also those who—compared to their counterparts—had their daily HWB more strongly (and positively) affected by previous peaks in SRN_DL. In other words, those young adults who felt particularly and consistently capable of dealing with their negative emotions also had a higher‐than‐usual level of well‐being the day after.

The persistence of SRN_DL (i.e., the within‐person carry‐over effect) over 21 days represents a lingering effect in which participants with high persistence of their SRN_DL perturbations do not easily return to their equilibrium (i.e., their general level of SRN), likely capturing people's perception of *enhanced* (higher‐than‐usual) *proactive control* over their daily negative emotions (Bandura 2001). Hence, it might not be so surprising that those young adults with prolonged peaks in their perceived capability to manage their anger and sadness are also the ones who benefit more from their SRN_DL to experience higher‐than‐usual levels of happiness and life satisfaction (i.e., their HWB). Moreover, these ‘enduring peaks’ might also reflect the onset of potential mean‐level changes in people's SRN_DL that, in the long run, might be responsible for future long‐term modification at the between‐person level (Hamaker 2012). Future long‐term studies are needed to ascertain such hypotheses (e.g., Neubauer et al. 2023).

Importantly, we found that the within‐person effect of SRN_DL on later HWB was statistically significant for those adults with high carry‐over effects of SRN_DL not simply while controlling for the time‐invariant covariates at the between‐person level (Rohrer and Murayama 2023) but also while taking into account the within‐person daily correlations among SRN_DL, HWB and negative daily events as well as by partialling out the (negative) spill‐over effect from negative daily events to later HWB. Hence, regardless of the number of daily negative events related to the participants' work/study environment, economic situation and health, reporting these kinds of daily events more than usual on a specific day predicted lower levels than usual of HWB the subsequent day. Although this result is in line with the literature suggesting that negative life events can decrease well‐being (Kettlewell et al. 2019), it is important to emphasise that the within‐person association between SRN_DL and N_DLE highlights how these variables are related: while it is not possible to directly influence daily negative events, increasing and maintaining SRN_DL beliefs related to these events is certainly a beneficial factor for HWB of young adults.

## 6 Limitations and Future Directions

Despite several strengths (e.g., intensive longitudinal data collected from young adults, a rigorous data analytical approach and a high response rate) our study shows some limitations. First, we considered only HWB as a measure of well‐being. Future studies should investigate also the relations between SRN_DL and other kinds of well‐being (e.g., eudaimonic well‐being; Ryff and Keyes 1995). Second, we focused on an Italian young adult sample. Hence, our results should be corroborated in samples from different cultures and/or across different developmental phases (e.g., during adolescence and the elderly). Third, our study investigated the relations between SRN_DL and HWB using a daily framework. Future research might benefit from using different time lags to ascertain whether the strength of the effect of SRN_DL on HWB could vary across shorter (e.g., hours) or longer (weekly) time frames. Fourth, in our study, we took into account only SRN_DL without considering other relevant facets of the broader emotion regulatory domain such as people's capability to rely on memories of positive emotional experiences (Gerbino et al. 2018) and/or interpersonal emotion regulation strategies (López‐Pérez et al. 2024). Future studies are needed to ascertain the extent to which people's self‐efficacy beliefs in these related‐yet‐different domains could additively contribute to sustaining individuals' well‐being, Fifth, the present study focused on a non‐clinical sample. For instance, we believe that investigating the relation between SRN_DL and HWB among participants with mood disorders could inform clinicians about the relevance of promoting SRN_DL in clinical settings. Finally, we recognise the exploratory nature of this study and acknowledge that our main effect—the persistence of SRN_DL in predicting the spillover from SRN_DL to HWB—is underpowered. Therefore, we recommend that future studies replicate this research with a larger sample.

## 7 Conclusion

Notwithstanding these limitations, our findings provide further empirical evidence about the importance of Bandura's Social Cognitive Theory in everyday life. (Bandura 2001). Feelings of anger and despondency are potentially ubiquitous in people's daily experiences. Helping individuals feel capable of managing such negative emotions and dealing with the situation/contexts that could trigger them is an important step to allowing people to take control—at least in part—over their well‐being. At the practical level, since mastery experience is the most important source of building self‐efficacy (Bandura 1997), researchers might consider designing interventions that expose individuals to controlled anger‐ or despondency‐inducing situations. Such interventions could help individuals develop their agentic control enhancing their ability to manage similar challenges during daily life.

## Author Contributions

**Antonio Zuffianò:** conceptualization, investigation, data curation, formal analysis, writing – original draft, writing – review and editing. **Fulvio Gregori:** conceptualization, investigation, data curation, formal analysis, writing – original draft, writing – review and editing. **Lucia Manfredi:** conceptualization, investigation, data curation, formal analysis, writing – original draft, writing – review and editing. **Elisabetta Beolchini:** writing – original draft, writing – review and editing. **Silvia Caldaroni:** conceptualization, investigation, data curation, formal analysis, writing – original draft, writing – review and editing. **Alessia Teresa Virzì:** writing – original draft, writing – review and editing. **Noemi Di Brango:** writing – original draft, writing – review and editing. **Virginia Isabel Barrero Toncel:** writing – original draft, writing – review and editing. **Stefania Sette:** writing – review and editing. **Valentina Paz Quilodrán Fuentes:** writing – original draft, writing – review and editing. **Bernadette Paula Luengo Kanacri:** writing – original draft, writing – review and editing.

## Ethics Statement

All procedures performed in studies involving human participants were in accordance with the ethical standards of the institutional research committee at Sapienza University of Rome and with the 1964 Helsinki Declaration and its later amendments or comparable ethical standards.

## Consent

Informed consent was obtained from all individual adult participants included in the study.

## Conflicts of Interest

The authors declare no conflicts of interest.

## Supporting information


**Data S1:** ijop70123‐sup‐0001‐Supinfo.docx.

## Data Availability

Data and analysis scripts used for this article can be accessed at the following link: https://osf.io/vfyzj/?view_only=8dab6c94123240d2962a910330c276a6.

## Endnotes

^1^
We also have conducted a Monte Carlo simulation to assess whether the sample size used in our study was sufficient (i.e., at least 80% power) to replicate a significant variance of the spillover effect from SRN_DL to HWB (BETA2), as observed in the unconditional model (*σ* ^2^ = 0.280). The simulation results indicated that a replication study with the same sample size would have 100% statistical power to detect this effect. Furthermore, we performed an additional Monte Carlo simulation to evaluate whether our sample size was adequate to replicate the main effect—specifically, the persistence of SRN_DL (PHI1) in predicting the spillover from SRN_DL to HWB (BETA2)—with the same effect size (*b* = 0.330). The results revealed that this effect is underpowered at the conventional 80% treshold, as a replication with the same sample size would have only 57.5% statistical power. The MPlus output for all simulations is available at OSF (link: https://osf.io/vfyzj/?view_only=8dab6c94123240d2962a910330c276a6).

^2^
In conditional model 2.b, in which we controlled for the time‐invariant covariates age and gender, the effect of L_H_PHI1 on BETA2 remained statistically significant (*β* = 0.357; 95% CI: [0.041, 0.653]). We did not find any significant effect of gender and age on the intercepts of the variables considered. We also conducted a sensitivity analysis by re‐estimating the model considering only participants who provided at least data for 11 days to test whether the main effect remained stable. The effect was virtually identical. All MPlus outputs (including the syntaxes) are available at the following OSF link: https://osf.io/vfyzj/?view_only=8dab6c94123240d2962a910330c276a6.
