# Supplementary material for: Regulatory Emotional Self‐Efficacy and Hedonic Well‐Being in Daily Life
Source: Int J Psychol. 2025 Nov 17;60(6):e70123. doi: 10.1002/ijop.70123 (PMC12620929; doi:10.1002/ijop.70123)
Supplement: Supplementary file 1 — Data S1: ijop70123‐sup‐0001‐Supinfo.docx. [file IJOP-60-e70123-s001.docx]

**Supplementary Materials**

Following the first round of revisions, we conducted a series of additional statistical analyses in response to the reviewers' comments. In addition to the main analyses already discussed in the manuscript, the OSF repository includes the following folders, which contain the datasets and outputs of the supplementary analyses. All materials are available at the following link:<https://osf.io/vfyzj/?view_only=8dab6c94123240d2962a910330c276a6>.

To improve transparency and accessibility, we provide below a brief guide for readers interested in exploring these additional materials.

*Robustness Analysis*

To assess the robustness of our findings, we complemented the main analysis (based on the mean-split of PHI1 to define low vs. high levels) with additional checks. Specifically, we created groups based on the median, as well as using the 25th and 75th percentiles as cutoffs. Results using the median were consistent with those from the main analysis, likely due to the near equivalence of mean and median under the normal distribution of PHI1. However, when using the 25th and 75th percentiles, the effect of interest did not emerge. All models and outputs are available in this folder.

*Sensitivity Analysis*

To test the stability of the main effect—namely, the predictive role of PHI1 on the spillover from SRN_DL to HWB (BETA2)—we re-estimated the model considering only participants who provided data for at least eleven days. The effect remained virtually identical. A summary of this analysis is also reported in Footnote 1 on page 13 of the main manuscript.
 *Power Analysis*

We conducted Monte Carlo simulations to evaluate whether our sample size provided sufficient statistical power. First, we tested the ability to replicate the variance of the spillover effect from SRN_DL to HWB (BETA2) observed in the unconditional model (σ² = .280). The simulation showed 100% power with the current sample size. Second, in the conditional model, we examined the main effect of PHI1 predicting the spillover (BETA2), with an observed effect size of b = .330. This analysis revealed insufficient power (57%) to detect this effect with our current sample size. A third simulation was performed to determine the sample size needed to reach adequate power (~80%) for this effect: a sample of 90 participants, each with 21 days of data, would be required.
